# Chemical investigation of biological trace evidence; toxicological screening of waste residues obtained from DNA extraction processes

**DOI:** 10.1007/s00414-023-03119-6

**Published:** 2023-11-16

**Authors:** Domenico Di Candia, Gaia Giordano, Michele Boracchi, Paolo Bailo, Paola Primignani, Andrea Piccinini, Riccardo Zoja

**Affiliations:** Dipartimento Di Scienze Biomediche Per La Salute, Istituto Di Medicina Legale E Delle Assicurazioni, Università Degli Studi Di Milano, 20133 Milan, Italy

**Keywords:** Toxicology on DNA extraction residues, Toxicology on one blood drop, Toxicological and genetic analyses, Forensic toxicology

## Abstract

In a forensic scenario, if biological stains are found in very small quantities, these are usually collected for DNA analyses, considered essential for the forensic investigation and thus excluding possible investigations by other forensic disciplines as forensic toxicology. We developed an experimental study to evaluate the feasibility of analyzing DNA extraction residues obtained from DNA extraction procedures to perform toxicological analysis, with the aim to extract both genetic and toxicological information without affecting or compromising the genetic sample and/or DNA extraction. DNA extraction from four blood samples (fortified with 5 molecules of interest with a final concentrations of 1 µg/mL, 100 ng/mL, 10 ng/mL and 5 ng/mL, respectively) were analyzed with QIAGEN QIAmp® DNA Mini kit. Three waste residues collected from the DNA extraction were analyzed for the toxicological investigation via Solid-Phase Extraction and High-Performance Liquid Chromatography—Tandem Mass Spectrometry analyses (Thermo Scientific™ TSQ Fortis™ II Triple-Quadrupole Mass Spectrometer). The analytical investigation revealed that our analytes of interest were detected in two different residues of the DNA extraction procedure, allowing both genetic and toxicological analyses without affecting the DNA identification. At last, the experimental protocol was applied to a hypothetical case, with encouraging results and allowing the identification of our molecules of interest.

## Introduction

In forensic cases where biological matrices as blood or urine are found in very small quantities, DNA identification is essential and of primary importance, thus preventing the feasibility of other possible forensic investigations. Although toxicological analyses are also very important for forensic cases, when a little amount of blood is found on a crime scene one of the top priorities is DNA analysis.

Toxicological analyses can be of considerable value in the determination of a subject's biological profile or in shedding light on the cause and/or manner of death of the individual.

Therefore, the possibility of assessing DNA extraction and evaluating the toxicological profile of a subject even from a single drop of blood can be considered as a new challenge in the forensic field.

After an in-depth literature review, no studies emerged that could respond to the situation described above. There is only one case study reporting that dried bloodstains were collected from a surface using cotton swabs and both genetic and toxicological investigations were performed on those samples. However, the details of the procedure are not explained, and it is not specified whether only one swab was used for both DNA and toxicological analyses [1]. Other studies have shown that it is possible to combine two different forensic disciplines on paraffin-embedded blocks: these substrates have been used to perform both DNA [2] and toxicological investigations [3–6] as well as proteomics [7] and western-blot studies [8]. However, no studies reported the combination of DNA investigations and toxicological analyses when only a single sample is available. On these bases, we wanted to create an experimental study to evaluate the possibility of using DNA extraction residues to perform toxicological analysis, with the aim to extract both genetic and toxicological information without compromising the genetic sample and/or the genetic extraction procedure.

## Experimental protocol

**Fig. 1:**
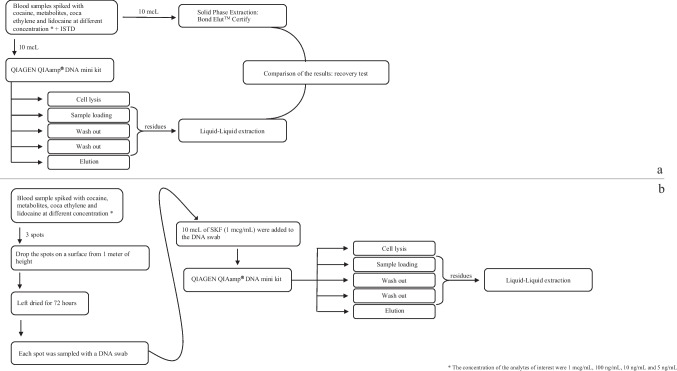
**a** Experimental procedure for toxicological analyses performed on three residues obtained from DNA extraction and comparison with standard SPE extraction. **b** DNA extraction from blood spots and toxicological analyses performed on residues obtained in steps nos. 2, 3 and 4 of DNA extraction procedure

Four blank whole blood samples were fortified with 5 molecules of interest with final concentrations of 1 µg/mL, 100 ng/mL, 10 ng/mL and 5 ng/mL respectively; the molecules involved in this study were cocaine, benzoylecgonine, ecgonine methyl ester, coca ethylene and lidocaine. Ten microliters of the spiked blood samples, together with 10 µL of our internal standard (IS) (SKF 525-A, 1 µg/mL), were processed with QIAGEN QIAmp® DNA Mini kit to perform DNA extraction. The genetic extraction procedure consisted in five steps: cell lysis, sample loading, first and second wash out and DNA elution. The sample loading and the washing steps consisted in three passages that produced waste residues, not involved in the subsequent steps of the DNA extraction procedures. These three passages were therefore selected and collected to perform toxicological analyses. DNA binds specifically to the QIAamp silica-gel membrane of the column, and it is separately removed afterwards, while contaminants pass through the column and are collected as waste of passage no. 2 (sample loading), or they are washed out during steps nos. 3 and 4 (first and second wash out). Therefore, these three DNA extraction residues were extracted via liquid–liquid extraction to perform toxicological investigations. Moreover, 10 µL of the four blood samples, previously fortified with our molecules of interest, were extracted via Solid Phase Extraction (gold standard in forensic toxicology) utilizing Bond Elut™ Certify cartridges with the aim to compare the results obtained with the results of liquid–liquid extraction performed on residues obtained from DNA extraction (Fig. 1a).

The second part of the experimental study consisted in recreating some blood stains using the samples previously spiked with our analytes of interest and dropping the samples on a surface; the spots were allowed to dry for 72 h and then collected with sterile cotton swabs. Subsequently, 10 µL of internal standard were added to the swab and the DNA was extracted with QIAGEN QIAmp® DNA mini kit. The residues obtained from the DNA extraction were analyzed with a liquid–liquid extraction procedure described in the material and methods section (Fig. 1b).

## Materials and methods

### Chemicals and Reagents

Analytical standards involved in this study as Cocaine Multi-component Mixture solution (250 µg/mL in acetonitrile, certified reference material Cerilliant®), and Lidocaine solution (1 mg/mL in methanol, certified reference material Cerilliant®), were purchased from Sigma-Aldrich and stored at -20 °C. Internal Standard (IS) SKF 525-A (Proadifen hydrochloride, analytical standard, > 95%, 100 mg) was purchased from Sigma-Aldrich as well. Working solutions of the analytical standards and IS were prepared in acetonitrile or methanol and stored at -20 °C until use. Solvents used in the extraction processes were purchased by PanReac AppliChem ITW Reagents (methanol, phosphate buffer solution pH 9 and phosphate buffer pH 6, 2-propanol, *n*-hexane, *n*-heptane, and water).

### Sample preparation

Four blank samples of whole blood were transferred to four lab test tubes where analytical Cocaine Multi-component Mixture standard solutions and lidocaine standard solutions were previously allowed to dry to obtain a final concentration of 1 µg/mL, 100 ng/mL, 10 ng/mL and 5 ng/mL for each analyte, in addition with 10 ng of our IS.

### DNA extraction

Ten microliters of each blood sample were spotted on a cotton swab and extracted with a QIAGEN QIAamp® DNA Mini kit using a QIAamp buccal swab spin protocol. All centrifugation steps were carried out at room temperature with a Thermo Scientific™ Micro CL 17 microcentrifuge. The microcentrifuge was utilized in the four steps of DNA purification (from the solution obtained from lysis to elution step) at 8000 and 13000 rpm.

DNA extraction was performed according to the QIAGEN QIAamp® DNA Mini kit manual: in the first step (lysis) the swab was placed into a microcentrifuge tube with 400 µL of Buffer AL (30% of guanine hydrochloride and 1% of maleic acid) and 20 µL of proteinase K. Subsequently, the sample was mix-pulsed for 15 s on a vortex mixer and incubated for at least 1 h at 56 °C and briefly centrifugated to remove drops from inside the lid. After the lysis, no precipitate was noted at the bottom of the microcentrifuge tube and 400 µL of ethanol 99% were added to the sample, mixed with a pulse-vortex for 15 s and, finally, briefly centrifuged. In the second step, the solution obtained was loaded in a QIAamp Spin Column and centrifuged at 6000 × g (8000 rpm) for 1 min. The QIAamp Mini Spin Column was placed in a clean 2 mL collection tube (provided in the kit) to initiate the washing steps. The third step consisted in a first wash out with 500 µL of AW1 buffer (a solution at 50% guanine hydrochloride in ethanol), while the fourth step was performed with 500 µL of AW2 buffer (Tris hydroxymethyl-aminomethane) to perform a second washing of the sample in ethanol. After each wash step, the column was centrifuged at 6000 × g (8000 rpm) for one minute and the collection tube containing the filtrate was discarded. The collection tubes in which the remains of the lysed sample and the waste of the washing steps were collected for the subsequent toxicological study. The fifth and last step was DNA elution assessed with 30 µL of buffer AE (10 mM Tris hydroxymethyl-aminomethane with 0.5 mM EDTA pH 9) after 1 min of a full-speed centrifugation (Fig. 1a).

### Liquid–liquid extraction

Toxicological analyses were performed on residues of DNA extraction obtained from step no. 2 and from the wash out residues of steps nos. 3 and 4 (see Fig. 1a). This procedure was applied on each blood sample involved in the study. Those three steps were selected for the analyses considering that they produce waste residues not involved in the subsequent phases of DNA extraction.

The residues were extracted with a liquid–liquid extraction technique. Thus, the samples were diluted with 5 mL of buffer phosphate pH 9 and extracted with 5 mL of chloroform/isopropanol/*n*-heptane (50:33:17; v/v). Samples were then positioned on a rotating wheel (Falc F205) for 30 min, and subsequently separated via centrifugation for 10 min at 3500 rpm (Thermo Scientific, Heraeus Biofuge Primo centrifuge). After the separation of the two phases obtained, the organic phase was collected and left drying under a gentle nitrogen stream. The three specimens (per blood sample analyzed) were reconstituted with 50 µL of methanol and 2 µL of the final solutions were analyzed with a Thermo Scientific™ TSQ Fortis™ II Triple-Quadrupole Mass Spectrometer (Fig. 1a).

### Solid-Phase extraction

Ten microliters of each blood sample, previously spiked with the above-mentioned molecules of interest and diluted in 5 mL of pH 6 buffer phosphate, were extracted with Solid Phase Extraction technique using Bond Elut™ Certify 130 mg (Agilent). Cartridges were conditioned with 3 mL of methanol and 3 mL of pH 6 phosphate buffer. Samples were centrifuged and the supernatant was loaded into the cartridge. Cartridges were then washed out with 3 mL of pH 6 phosphate buffer and 0.5 mL of methanol and subsequently led to exsiccation. Eluting solution was composed by 2 mL of ethyl acetate/ammonium hydroxide (98:2, v:v) and 2 mL of methanol/ammonia solution (98:2, v:v). Eluates were exsiccated and then reconstituted with 50 μL of methanol (Fig. 1a).

### Hypothetical real sample preparation

As a last step, three drops of the blood samples were dropped on a surface and were allowed to dry for 72 h. Each spot was then sampled with a different cotton swab, one for each drop. Ten microliters of Internal Standard (IS) were added to the swabs and then used for DNA extraction with QIAGEN QIAamp® DNA Mini kit following the procedure previously described. At the end, the three residues of DNA extraction obtained from passages nos. 2, 3 and 4 were collected for toxicological analyses with liquid–liquid extraction procedure as reported before (Fig. 1b).

### Instrumental conditions

The Thermo Scientific™ TSQ Fortis™ II Triple-Quadrupole Mass Spectrometer (Thermo Scientific, San Jose, CA, U.S.A.) was associated to a HPLC system constituted by a Surveyor MS quaternary pump with degasser, Surveyor AS auto-sampler, oven with Rheodyne valve, 20 µL loop and with a heated electrospray ionization source (HESI). The chromatographic column used was a reverse phase Thermo Scientific Zorbax Eclipse XDB-C18 4.6 × 50 mm, with particle size 1.8 μm, stabilized at 35 °C and with a constant flow rate of 0.600 mL/min. Twenty millimolar ammonium formate in water and methanol were the solvents that constituted the mobile phase for the analyses. The gradient of the mobile phase was set for solvent A at: 90% in the first minute, decreased to 15% at the fourth minute and maintained at this percentage until minute 6, at the end was brought back to starting conditions from minute 6 to minute 10. The capillary and vaporization temperature were set at 330 and 280 °C respectively. Electrospray tension (with positive mode) and positive ion spray voltage were set at 3.5 kV. Sheath gas, aux gas and sweep gas were set at 45, 20 and 10 Arb, respectively. CID gas was set at 1.5 mTorr, Q1 resolution was selected at 0.4 FWHM and Q3 resolution was 0.7 FWHM. The Resolution power of Full Size was 70.000 FWHM. The mass range was between 50 to 650 m/z. ﻿Automatic Gain Control was set at 5 × 10^−4^ with a maximum injection time of 100 ms. Quadrupole filtered precursor ions had an isolation range of 2 m/z.

### Identification criteria for qualitative confirmation

All the samples under investigation were screened with a customized inclusion list containing our molecules of interest and the IS. The molecules under investigation were confirmed following international standard guidelines for forensic toxicology and assessed via reference material (analytical standards) [9, 10]. The parent ion, product ions, retention time and signal-to noise ratio of each molecule are reported in Tables 1 and 2.

**Table 1:** Identification criteria for molecules under investigation

Molecule	Parent ion (m/z)	Product ion (m/z)			Retention time
Cocaine	304.30	182.10	150.00	105.10	4.55
Benzoylecgonine	290.00	168.00	105.00	76.90	4.54
Ecgonine methyl ester	200.16	282.00	90.00	82.00	0.73
Coca ethylene	318.20	196.00	108.00	82.00	4.78
Lidocaine	235.20	86.20	58.10	120.10	4.30
SKF-525 A	354.50	209.10	167.10	91.00	5.81

**Table 2:** Signal-to-noise ratio of samples analyzed

S/N ratio								
Molecule	1 µg/mL		100 ng/mL		10 ng/mL		5 ng/mL	
	3^rd^ step	4^th^ step	3^rd^ step	4^th^ step	3^rd^ step	4^th^ step	3^rd^ step	4^th^ step
Cocaine	350	371	151	152	8	9	< LOD	< LOD
Benzoylecgonine	402	373	221	202	5	6	< LOD	< LOD
Ecgonine methyl ester	298	311	111	171	6	4	< LOD	< LOD
Coca ethylene	441	461	266	288	9	8	< LOD	< LOD
Lidocaine	231	256	100	114	7	8	< LOD	< LOD
SKF-525 A	501	482	493	423	520	519	466	512

The table is divided into the scaling dilution chosen for this study (1 µg/mL, 100 ng/mL, 10 ng/mL, 5 ng/mL)

### Quantitative confirmation

Linear calibration curves were prepared starting from working solutions with the following ranges: 1–5-10–20-40–80 ng/mL and the coefficient of determination (r^2^) for linear calibration model was calculated ≥ 0.99 for each molecule.

The carryover effect was investigated by injecting in triplicates an extracted blank sample of whole blood after the highest calibration points. No carryover could be noted.

LOD (Limit Of Detection) was determined as the lowest concentration with a signal-to-noise (S/N) ratio of the peak areas ≥ 3 (0.3 ng/mL) and with all the acceptance criteria met (retention time, peak shape, mass spectral ion ratio. LLOQ was determined as the lowest non-zero calibration point in which all criteria were met (detection, identification, bias, and precision) and S/N ratio of the peak areas ≥ 10 (1 ng/mL) [9, 10].

## Results

DNA was successfully extracted with the QIAGEN QIAamp® DNA Mini kit procedure mechanically positioned on swabs for the experimental procedure. Toxicological analyses performed on residues of step 2, 3 and 4 of the DNA extraction procedure showed positive findings in steps nos. 3 and 4. The first wash (step no. 3) was performed with a solution at 50% guanine hydrochloride (AW1) and the second one (step no. 4) was carried out with tris hydroxymethyl-aminomethane (AW2). The characterization of the molecules under investigation was possible in three out of four blood samples analyzed. Indeed, the characterization of the molecules was not achievable in the blood specimen fortified with 5 ng/mL of our analytes of interest, in which the signal-to-noise ratio was below the Limit of Detection of the instrument. The characterization of the molecules was permitted in the other blood samples involved in the study, but the quantification was not allowed in one blood sample (precisely the specimen fortified with 10 ng/mL of the molecules under investigation; as reported in Fig. 4) due to the fact that the signal-to-noise ratio was below the Limit of Quantification of the instrument.

**Fig. 2:**
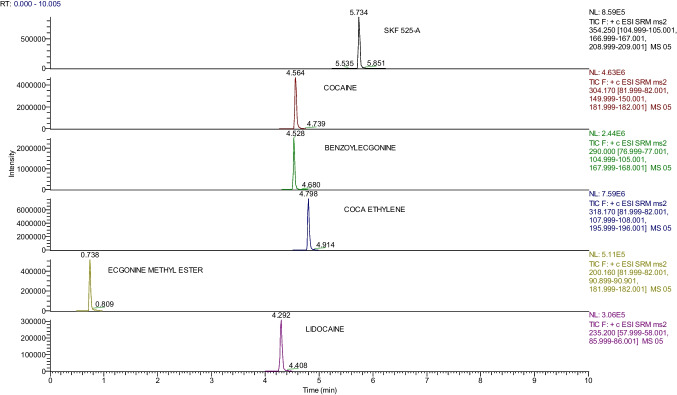
Chromatographic spectra of molecules and internal standard (concentration 1 µg/mL) detected in the residue obtained from the third passage of DNA extraction protocol

**Fig. 3:**
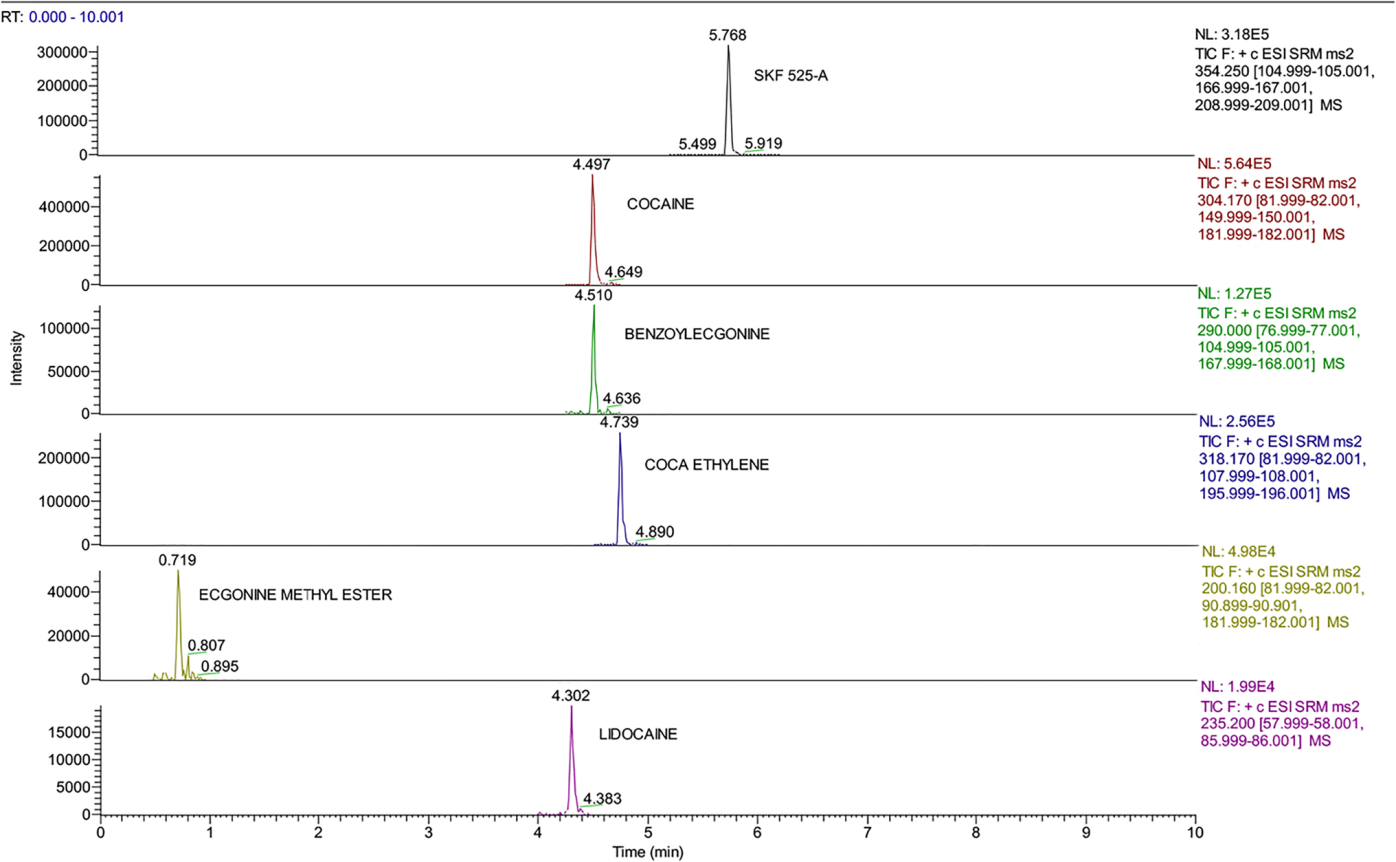
Chromatographic spectra of molecules and internal standard (concentration 100 ng/mL) detected in the residue obtained from the third passage of DNA extraction protocol

**Fig. 4:**
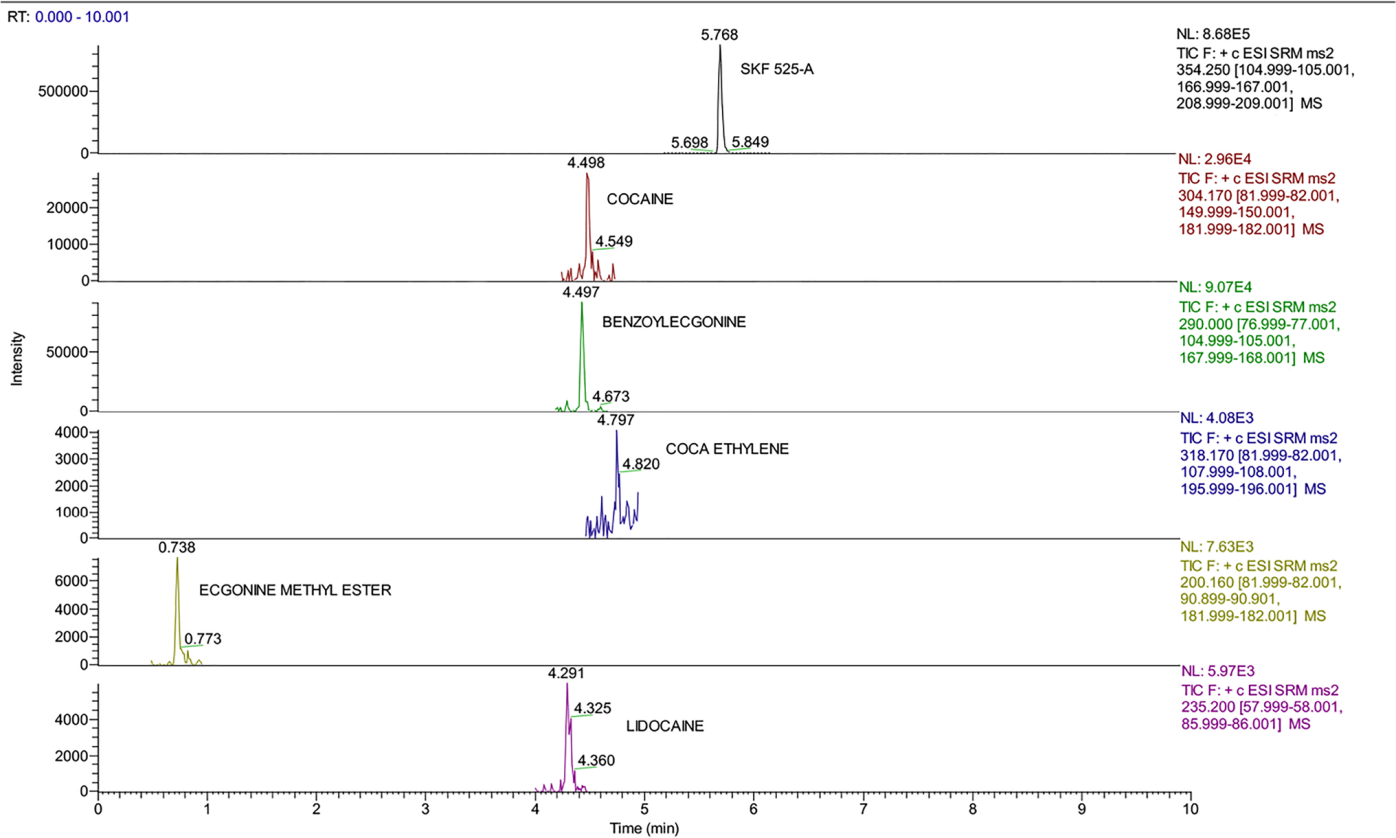
Chromatographic spectra of molecules and internal standard (concentration 10 ng/mL) detected in the residue obtained from the third passage of DNA extraction protocol

Examples of chromatographic spectra obtained for each molecule detected in the third step of DNA extraction are reported in Figs. 2, 3, and 4.

### Comparison between SPE technique and L-L extraction

The blood sample previously fortified with the molecules of interest was extracted with Solid-Phase extraction technique. Bond Elut™ Certify Agilent (130 mg) cartridges were chosen for the extraction procedures and the extraction protocol was reported in the material and methods section of this paper. In Tables 3, 4, 5, and 6 were reported the absolute recoveries of the analytes detected with SPE extraction and L-L extraction. The comparison was performed with the response of the analytes of interest in neat aqueous solution at 1 µg/mL, 100 ng/mL, 10 ng/mL and 5 ng/mL [9, 10] (Tables 3, 4, 5, and 6). The results obtained from recovery test could be considered satisfactory, as the gold standard SPE extraction was confirmed to have higher molecule recoveries, even if the liquid–liquid extractions performed on the DNA residues also gave excellent results. Moreover, the %CV (coefficient of variation) remained below %CV < 10 in each molecule. The results obtained from steps nos. 3 and 4 were evaluated together to highlight the potential of total extractive procedure (of both steps) with the extractive efficiency of the gold standard SPE extraction. The extractive efficiency was evaluated with absolute recovery tests.

**Table 3:** Comparison of recovery test (recovery %) and coefficient of variation (%CV) obtained from Solid-Phase extraction (SPE extraction) of 10 µL of blood sample (fortified at 1 µg/mL) and liquid–liquid extraction (LL extraction) of third and fourth residues of DNA extraction

	SPE extraction		LL extraction on residues nos. 3 and 4 of DNA extraction	
Molecules	Recovery %	% CV	Recovery %	% CV
Cocaine	92	7.57	74	8.77
Benzoylecgonine	85	5.72	69	6.64
Coca ethylene	75	7.69	71	7.34
Ecgonine methyl ester	88	6.74	76	7.47
Lidocaine	82	9.52	72	8.53

**Table 4:** Comparison of recovery test (recovery %) and coefficient of variation (%CV) obtained from Solid-Phase extraction (SPE extraction) of 10 µL of blood sample (fortified at 100 ng/mL) and liquid–liquid extraction (LL extraction) of third and fourth residues of DNA extraction

	SPE extraction		LL extraction on residues nos. 3 and 4 of DNA extraction	
Molecules	Recovery %	% CV	Recovery %	% CV
Cocaine	91	6.51	70	8.08
Benzoylecgonine	82	7.01	62	6.33
Coca ethylene	77	5.99	71	5.89
Ecgonine methyl ester	82	7.11	70	6.67
Lidocaine	84	8.23	68	7.32

**Table 5:** Comparison of recovery test (recovery %) and coefficient of variation (%CV) obtained from Solid-Phase extraction (SPE extraction) of 10 *µ*L of blood sample (fortified at 10 ng/mL) and liquid–liquid extraction (LL extraction) of third and fourth residues of DNA extraction

	SPE extraction		LL extraction on residues nos. 3 and 4 of DNA extraction	
Molecules	Recovery %	% CV	Recovery %	% CV
Cocaine	84	6.73	/	/
Benzoylecgonine	79	6.02	/	/
Coca ethylene	80	7.57	/	/
Ecgonine methyl ester	86	6.86	/	/
Lidocaine	80	7.77	/	/

**Table 6:** Comparison of recovery test (recovery %) and coefficient of variation (%CV) obtained from Solid-Phase extraction (SPE extraction) of 10 *µ*L of blood sample (fortified at 5 ng/mL) and liquid–liquid extraction (LL extraction) of third and fourth residues of DNA extraction

	SPE extraction		LL extraction on residues nos. 3 and 4 of DNA extraction	
Molecules	Recovery %	% CV	Recovery %	% CV
Cocaine	89	5.43	/	/
Benzoylecgonine	82	6.00	/	/
Coca ethylene	78	7.54	/	/
Ecgonine methyl ester	80	5.55	/	/
Lidocaine	81	9.06	/	/

In Tables 5 and 6, the recovery test and coefficient of variation of our analytes of interest at 10 ng/mL and 5 ng/mL were reported for the SPE extraction technique only, due to the inability of quantitate the molecules at 10 ng/mL and to not characterized the molecules in the concentration even lower.

### Real samples results

**Fig. 5:**
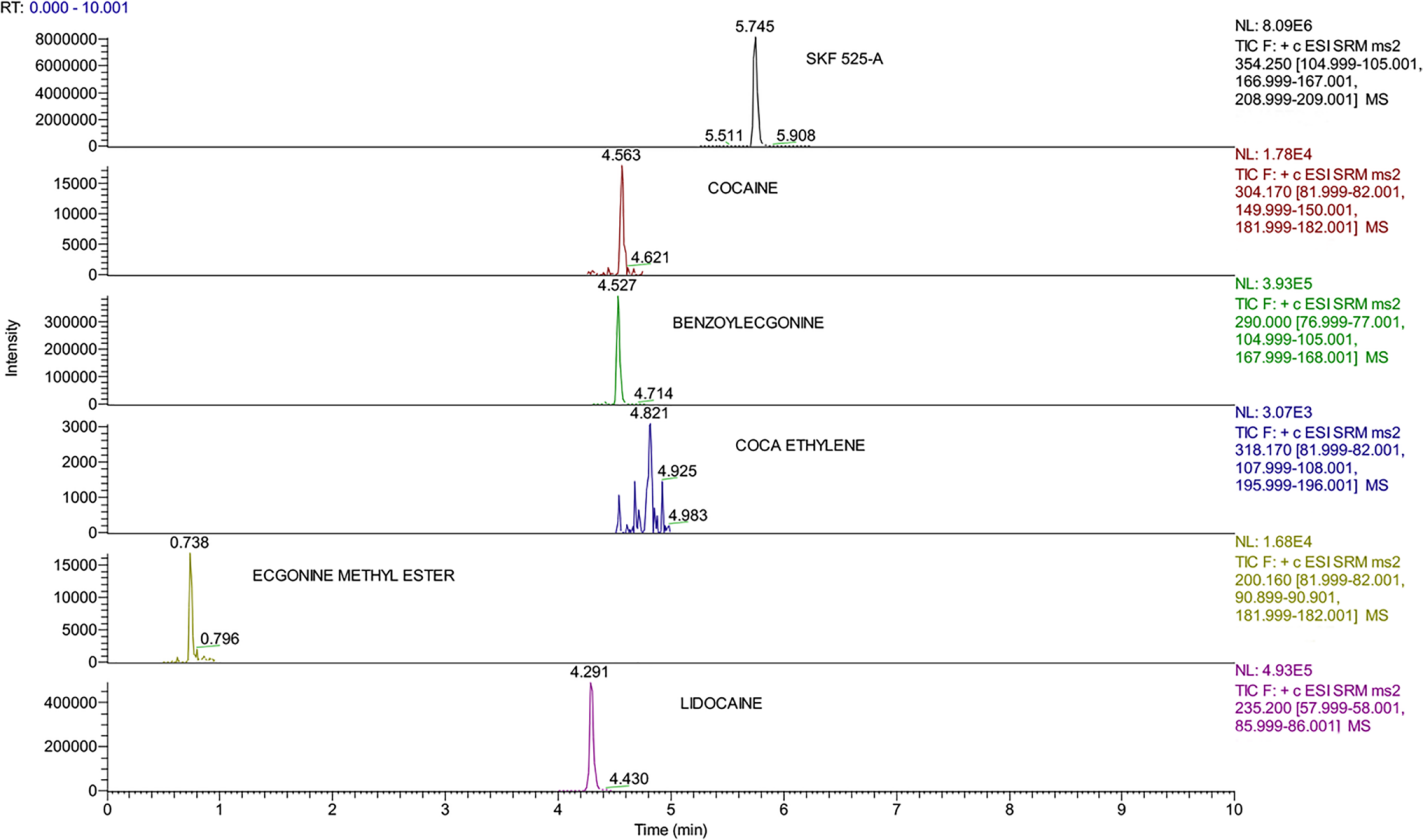
Chromatographic spectra of our analytes of interest and internal standard detected in residue obtained from the third step of DNA extraction protocol (simulation of real case)

**Fig. 6:**
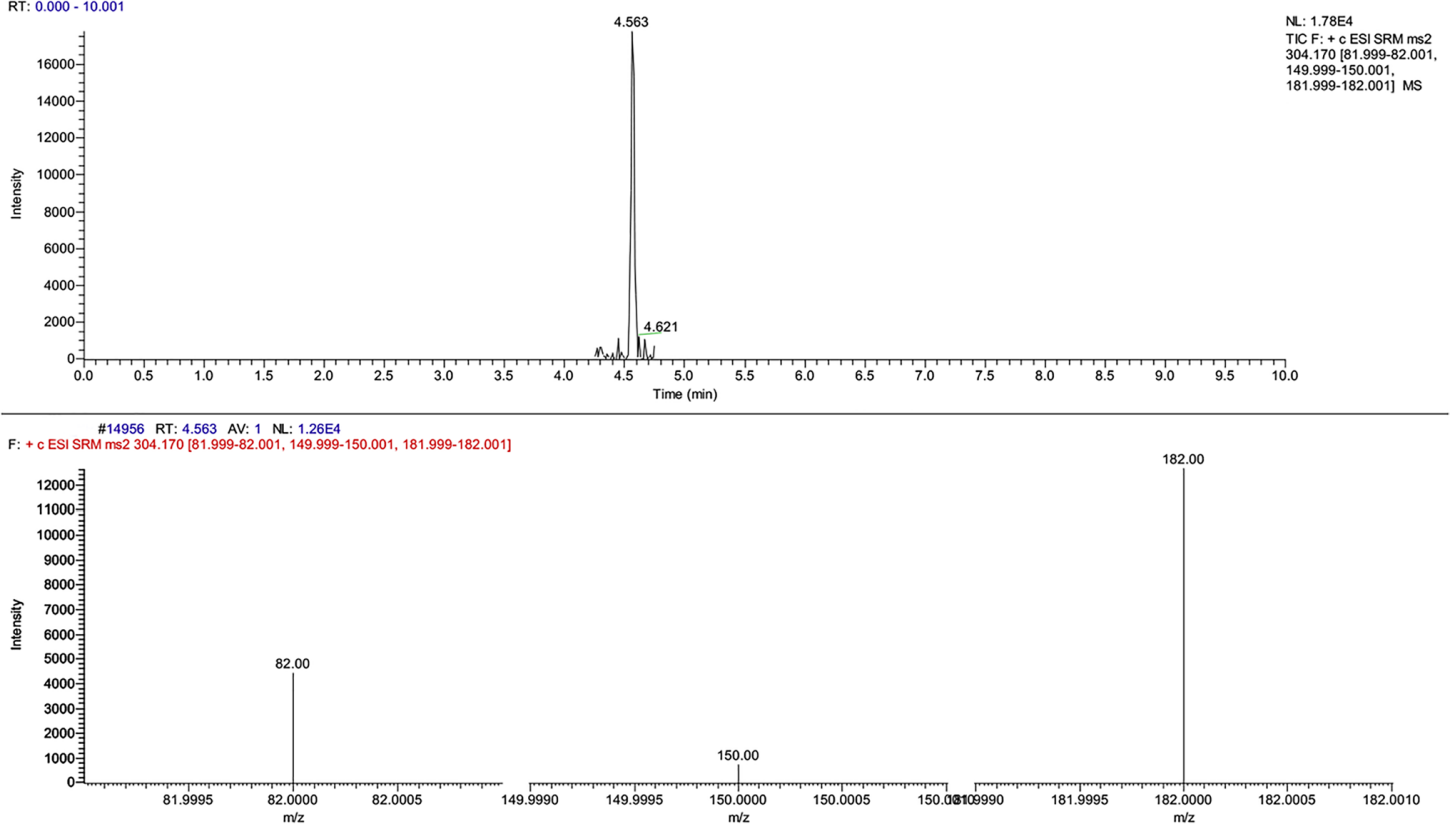
Chromatographic representation of cocaine and its mass spectral ion ratio (simulation of real case)

**Fig. 7:**
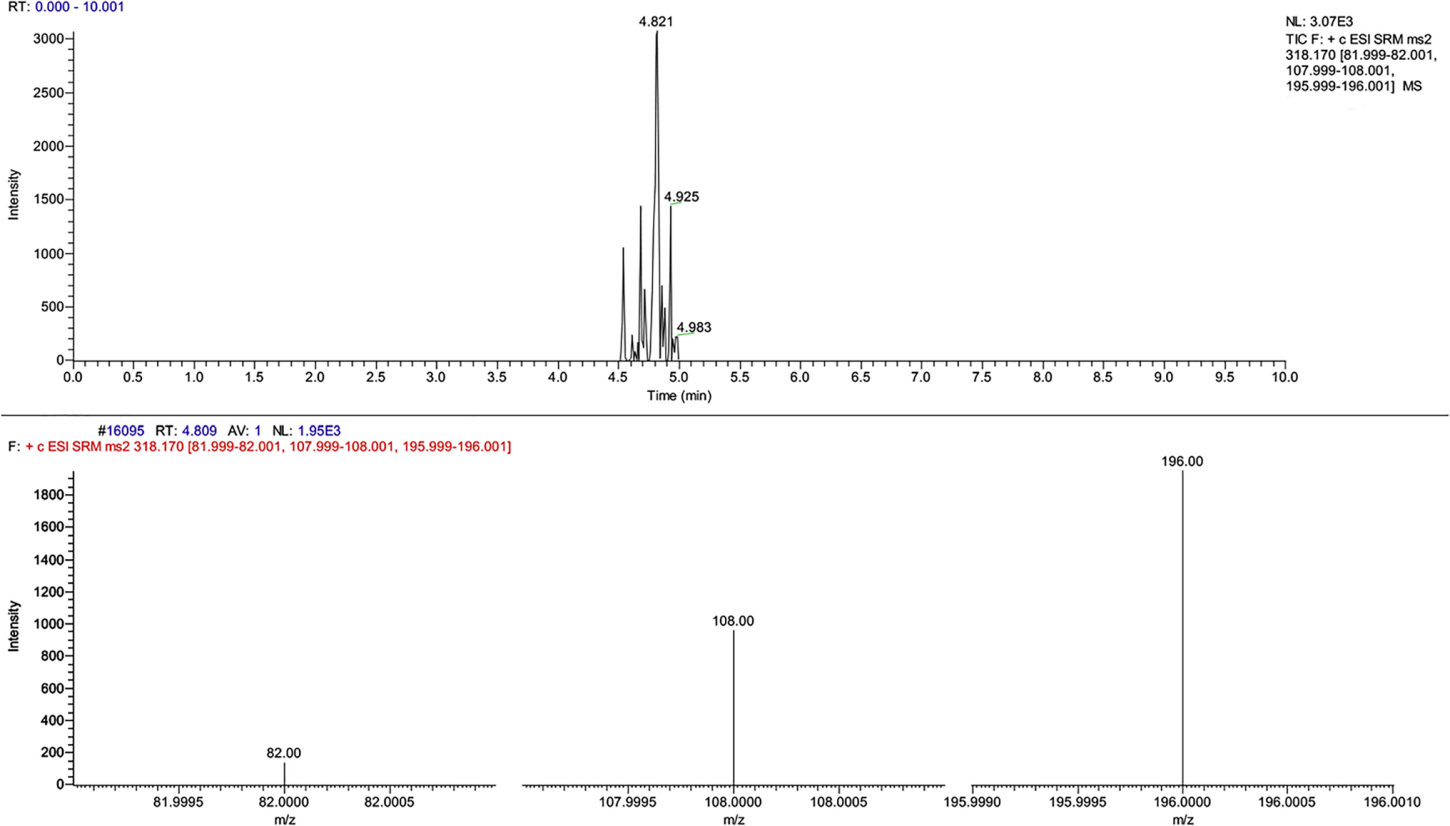
Chromatographic representation of coca ethylene together with its mass spectral ion ratio (simulation of real case)

DNA was successfully extracted with the QIAGEN QIAamp® DNA Mini kit procedure from the dry blood spots collected with swabs (the blood samples analyzed were fortified at 1 µg/mL, 100 ng/mL and 10 ng/mL; the blood sample spiked with our analytes of interest at 5 ng/mL was excluded from the second part of the experimental procedure due to the difficulties in characterizing the molecules in question, as seen Table 2 and 6). The analyses performed on the blood spots gave positive results for residues collected at steps nos. 3 and 4, while no positive results were obtained from step no. 2. Figure 5 reports the chromatographic spectra of our molecules of interest and internal standard detected at step no. 3. Moreover, in Figs. 6 and 7, we can appreciate the characteristic mass spectral ions ratio of cocaine and coca ethylene detected at step no. 3.

## Discussion

In this work, we developed an experimental study with the purpose of using DNA extraction waste residues as matrices to perform toxicological analysis in forensic cases with the aim to extract both genetic and toxicological information from small quantities of biological samples, without affecting or compromising the DNA sample. Thus, we developed a protocol for extracting DNA with the QIAGEN QIAmp® DNA Mini kit from four blood samples spiked with five molecules of interest (cocaine, benzoylecgonine, coca ethylene, ecgonine methyl ester and lidocaine) at different concentration (1 µg/mL, 100 ng/mL, 10 ng/mL and 5 ng/mL). During DNA extraction, 3 steps produce waste residues, not involved in the subsequent steps of DNA extraction. These three waste residues were used for toxicological investigation through a liquid–liquid extraction procedure. DNA binds specifically to the QIAamp silica-gel membrane of the column whereas contaminants (in our specific case the analytes added to the blood sample) pass through the column and could be collected as waste of phase no. 2 (sample loading) or they can be washed out during steps nos. 3 and 4 (first and second wash out steps).

From the experimental study, the toxicological analyses gave positive results in waste residues collected from steps nos. 3 and 4 of the DNA extraction procedure in the blood samples analyzed, while no positive results were obtained from the waste residue of sample loading step. At the end, from the toxicological results both qualitative and quantitative confirmation was permitted for all the molecules investigated in blood samples fortified at 1 µg/mL and 100 ng/mL. The characterization of the molecules was possible at the concentration immediately lower but was not evaluable at the lowest concentration considered in the study. Moreover, the experimental protocol gave satisfactory results in recovery test if compared to the samples extracted with SPE procedure, demonstrating the possible application of toxicological investigations on DNA extraction residues. Finally, considering the results obtained from the real case simulation (Fig. 5), we demonstrated that the molecules were successfully qualitatively detected. In conclusion, the purpose of this study was to qualitatively, and when possible quantitatively, investigate the feasibility of both extraction techniques and, also considering that we could not be certain of the actual amount of blood that was sampled during swabbing procedures, quantitation was voluntarily not assessed. Identification was possible also for cocaine and coca ethylene, in which the signal-to-noise ratio was 110 and 153 respectively, (cocaine mass spectral ions ratio was respected and maintained along the entire peak (Fig. 6); coca ethylene mass spectral ions ratio was respected and maintained as well (Fig. 7)).

## Conclusion

In conclusion, this study has successfully applied toxicological investigations to DNA extraction waste residues. Our work demonstrated that waste products derived from genetic analyses should not be considered as useless but could be employed for toxicological purposes as it was possible to qualitatively determinate the presence of analytes previously added to a blood sample and then extracted via Qiagen DNA Mini kit.

Our method can be considered as a valid opportunity for toxicological screening on extremely small biological sample allowing the analyses of both DNA and molecules of toxicological interest.

We could consider this study as a pioneering work in this field: lower concentrations and different analytes will be investigated with the purpose of optimizing this extraction technique and test the limits of this new method.

## Data Availability

The datasets generated during and/or analyzed during the current study are available from the corresponding author on reasonable request.
